# Comparison of pre-operative computed tomography cholangiography and intraoperative cholangiography in laparoscopic cholecystectomy: a retrospective analysis

**DOI:** 10.1186/s12893-023-02089-1

**Published:** 2023-06-30

**Authors:** Douglas Chung

**Affiliations:** Campbelltown Hospital, Therry Road, Campbelltown, NSW 2560 Australia

## Abstract

**Background:**

The role of intra-operative cholangiography (IOC) in laparoscopic cholecystectomy (LC) is controversial. CT cholangiography (CTC) provides a reliable assessment of biliary anatomy, potentially reducing operating times, open conversion, and complication rates. This study aims to assess the safety and effectiveness of routine pre-operative CTC.

**Methods:**

A single centre retrospective analysis was undertaken of all elective laparoscopic cholecystectomies between 2017 and 2021. Information was obtained from a general surgical database alongside hospital electronic medical records. T-tests and Chi^2^ tests were used to assess statistical significance.

**Results:**

Of 1079 patients, 129 (12.0%) underwent routine pre-operative CTC, 786 (72.8%) routine IOC, and 161 patients (14.9%) neither modality. Comparing CTC and IOC, the CTC group had higher rates of open conversion (3.1% vs. 0.6%, p 0.009), subtotal cholecystectomies (3.1% vs. 0.8%, p 0.018), and length of stay (1.47 vs. 1.18 nights, p 0.015). Comparing the prior groups together against those utilising neither modality, the latter had reduced operative time (66.29 vs. 72.47, p 0.011), but increased rate of bile leak (1.9% vs. 0.4%, p 0.037) and bile duct injury (1.2% vs. 0.2%, p 0.049). Co-dependence between operative complications was noted in linear regression.

**Conclusion:**

Biliary imaging with either CTC or IOC is beneficial in reducing bile leak and bile duct injury, and its routine use LC is recommended. However, routine CTC is inferior to routine IOC in preventing conversions to open surgery and subtotal cholecystectomy. Further research may be undertaken to evaluate criteria for a selective CTC protocol.

## Introduction

The role of Intra-operative cholangiography (IOC) in laparoscopic cholecystectomy (LC) is controversial. Its proponents cite its ability to identify aberrant biliary anatomy [1], consequently reducing incidence of bile duct injuries (BDI), as well as identification of retained bile duct stones as the reasons for its continued use [2–4]. Detractors have noted lack of proven cost effectiveness, prolonged operating times, and lack of efficacy [5–7]. Not infrequently, intra-operative difficulties can limit a surgeon’s ability to safely perform IOC, from significant fibrosis to oedema. With improvements in image quality and radiation exposure, CT cholangiography (CTC) is able to provide reliable and detailed assessment of biliary anatomy prior to commencing the operation. Its purported benefits include shorter operative time, reduced rates of open conversion, and reduced incidence of post-operative complications [8]. However, data on its effectiveness and performance relative to routine IOC is lacking. As such, this study aims to retrospectively assess the safety and effectiveness of the practice of regular CTC in a metropolitan centre as a routine modality for biliary imaging in LC.

## Aims

### Primary objective

To assess the safety and effectiveness of CTC in comparison to routine IOC, comparing length of stay, operative time, and complication rates.

### Secondary objectives

To assess the relative safety and effectiveness of CTC and IOC against no patients not undergoing routine biliary imaging.

## Methods

A single centre retrospective analysis was undertaken of elective LC performed at a tertiary hospital between 2017 and 2021, following the introduction of routine CTC into standard practice. Data linkage was undertaken of information from the pre-existing General Surgical database and the hospital Electronic Medical Records (eMR) system. All surgeons undertaking LC did so in a uniform fashion, utilising Strasberg dissection where possible with identification of the critical view of safety. CTC was undertaken within 2 weeks of the procedure, with 3D reconstructions and films available with patients on arrival. IOC was performed using a Reddick-Olsen catheter and urografin.

IBM SPSS was utilised to perform statistical analysis, utilising T-tests and Pearson chi^2^ tests to assess statistical significance between variables. A literature review was undertaken utilising EMBASE with the terms ‘cholangiogram’ AND ‘laparoscopic cholecystectomy’, alongside Google scholar.

### Variables

Independent : Modality and timing of cholangiography.

Control : Patient demographics, background medical issues, pre-operative diagnosis.

Dependent : Length of stay, complications, representations, readmissions, and mortailities.

Exclusion : Concurrent major procedures, aborted procedures, planned open cholecystectomies, emergency procedures.

### Definitions

**Frailty** is defined using criteria factors obtained from the Charlson Comorbidity Index [9] not captured by previous criteria. These include malignancy, cognitive and functional deficits, immunosuppression, connective tissue diseases, and anaemia.

**Smoking** is defined as an individual who is actively smoking, or has had a previous smoking history in excess of 5 pack years.

**Alcohol** use is defined as an individual who drinks alcohol in excess of 1 standard drink daily, or has had complications associated with alcohol abuse.

## Results

**Fig. 1:**
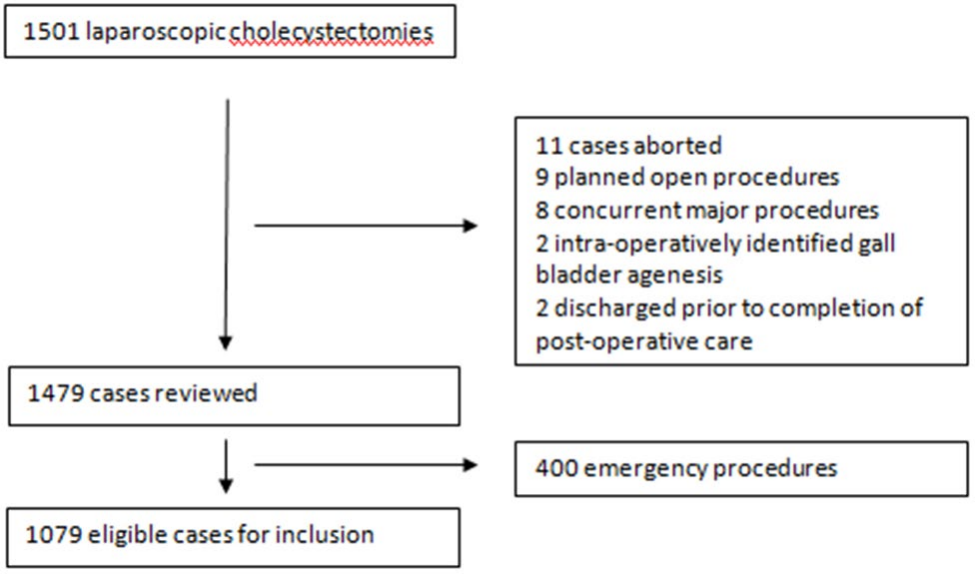
Case inclusion

### Patient demographics

A total of 1079 patients were eligible for review after exclusion, of which 129 (12.0%) underwent routine pre-operative CTC, whilst 786 (72.8%) underwent IOC. A further 161 (14.9%) did not undergo either imaging modality. No significant differences were identified in patient demographics or medical co-morbidities between the groups. Table 1 compares the demographics of patients between the group undergoing pre-op CTC and those that did not undergo CTC, whilst Table 2 breaks down the groups by proportion. Table 3 breaks down the indications for surgery between the various groups.

**Table 1:** Patient Demographics

	All (N = 1079)	Pre-op CTC (N = 129)	No pre-op CTC (N = 950)	p value
Age	47.04 ± 16.49	47.64 ± 16.35	46.95 ± 16.52	0.658
BMI	30.81 ± 6.98	31.11 ± 7.63	30.76 ± 6.88	0.608
Female gender	795 (73.7)	95 (73.1)	700 (73.8)	0.868
ASA	1.95 ± 0.66	1.94 ± 0.691	1.95 ± 0.66	0.846
Hypertension	259 (24.1)	33 (25.4)	226 (23.9)	0.709
Cardiovascular disease	90 (8.4)	13 (10.0)	77 (8.1)	0.472
Neurological disease	48 (4.5)	5 (3.8)	43 (4.5)	0.717
Coagulopathy	64 (5.9)	4 (5.8)	60 (6.3)	0.140
COPD	105 (9.8)	8 (6.2)	97 (10.3)	0.140
Diabetes mellitus	142 (13.2)	14 (10.8)	128 (13.5)	0.383
Liver disease	46 (4.3)	5 (3.8)	41 (4.3)	0.797
Kidney disease	15 (1.4)	1 (0.8)	14 (1.5)	0.517
Mental health disorder	137 (12.7)	15 (11.5)	122 (12.9)	0.663
Frailty	160 (14.9)	20 (15.4)	140 (14.8)	0.860
Smoking	297 (30.4)	41 (33.3)	256 (29.9)	0.444
Alcohol	271 (30.5)	31 (27.7)	240 (30.9)	0.485

**Table 2:** Imaging modality by proportion

Modality	No IOC	IOC	Pre-op CTC
Total	163	786	129
Proportion (%)	15.1	72.8	12.0

**Table 3:** Indications for surgery

	No IOC	IOC	Pre-op CTC
Abdominal pain	3 (1.8)	8 (1.0)	
Bilary colic	69 (42.3)	256 (32.6)	50 (38.7)
Cholelithiasis	52 (31.9)	292 (37.2)	50 (38.7)
Choledocholithiasis	5 (3.1)	45 (5.7)	5 (3.9)
Chronic cholecystitis	22 (13.5)	88 (11.2)	18 (14.0)
Previous cholangitis		10 (1.2)	
Polyps	6 (3.7)	23 (2.9)	1 (0.8)
Previous pancreatitis	4 (2.5)	49 (6.2)	3 (2.3)
Mirrizzi		1 (0.1)	
Dyskinesia			1 (0.8)
Sludge		3 (0.4)	
Others	3 (1.8)	11 (1.4)	1 (0.8)

### Post-operative course and complications

**Table 4:** Comparison of post-operative course

	Pre-op CTC (N = 129)	IOC (N = 786)	p value
Operative time (min)	68.89 ± 28.33	73.06 ± 28.54	0.123
Length of stay (nights)	1.47 ± 1.55	1.18 ± 1.54	**0.015**
Conversion to open	4 (3.1)	5 (0.6)	**0.009**
Subtotal resection	4 (3.1)	6 (0.8)	**0.018**
Bile duct injury	1 (0.8)	1 (0.1)	0.146
Post operative bile leak	1 (0.8)	3 (0.4)	0.533
Representation	3 (2.3)	38 (4.8)	0.198
Readmission	3 (2.3)	19 (2.4)	0.943
Return to theatres	1 (0.8)	7 (0.9)	0.892

**Table 5:** Complication rates comparing peri-operative imaging and patients with neither

	Biliary imaging (N = 915)	No biliary imaging (N = 161)	p value
Operative time (min)	72.47 ± 28.53	66.29 ± 26.65	**0.011**
Length of stay (nights)	1.22 ± 1.26	1.42 ± 1.39	0.073
Conversion to open	9 (1.0)	3 (1.9)	0.324
Subtotal resection	10 (1.1)	3 (1.9)	0.406
Bile duct injury	2 (0.2)	2 (1.2)	**0.049**
Post operative bile leak	4 (0.4)	3 (1.9)	**0.037**
Representation	41 (4.5)	6 (3.7)	0.672
Readmission	22 (2.4)	3 (1.9)	0.678
Return to theatres	8 (0.9)	3 (1.9)	0.248

**Table 6:** Linear regression p values

	Intent to undertake CTC	Intent to undertake biliary imaging
Conversion to open	0.164	0.862
Subtotal resection	0.380	0.938
Bile duct injury	0.682	0.588
Post operative bile leak	0.575	0.367

Comparison between groups undergoing CTC and IOC is presented in Table 4. There was a higher rate of conversions to open procedure in the CTC group at 3.1% relative to the IOC group at 0.6% (p 0.009). Rates of subtotal cholecystectomies were also raised at 3.1% relative to 0.8% (p 0.018). Additionally, length of stay in hospital increased from an average of 1.18 nights in the IOC group to 1.47 nights in the CTC group (p 0.015). No statistically significant benefit was identified in undertaking pre-operative CTC. Rates of readmission, representation, return to theatres, bile duct injuries, and post-operative bile leaks were comparable between groups.

The group undergoing biliary imaging, combining the IOC and CTC groups, is compared to the group not undergoing any form of biliary imaging in Table 5. Those not undergoing biliary imaging noted a reduction in operating time from 72.47 min to 66.29 min (p 0.011). However, rates of post-operative bile leak increased from 0.4 to 1.9% (p 0.037), while rates of bile duct injury increased from 0.2 to 1.2% (p 0.049).

A linear regression analysis was undertaken of the procedural complications in Table 6. In comparing CTC and IOC, there is loss of significance in factors when all post-operative complications are accounted for. Similar findings are noted in comparing those not undergoing biliary imaging compared to those doing so. Both these findings suggest a significant degree of co-dependence between the variables.

The instances of BDI that occurred were classified using the Bismuth Strasberg classification. One instance of a type A injury was missed BDI noted with CTC. One incidence of a type D injury was identified intrao-peratively, and confirmed utilising an IOC. Two instances of type A injuries occurred where neither CTC or IOC was utilised.

**Table 7:** Reasons for inability to perform IOC

Reason for failure to perform IOC	Number
Conversion to open	8
Technically challenging	36
Contrast allergy	4
Equipment failure	6
Total	54

Table 7 examines the reasons for inability to perform or complete IOC. 54 occurrences were identified. Reasons include including technical difficulties, equipment failure, and contrast allergies.

No deaths were identified as a direct consequence of these procedures.

## Discussion

### Utilisation and choice of peri-operative imaging

There is evidence in the acute setting that CTC may be utilised to grade the risk of conversion to open surgery [13], but this is untested in an elective setting. Our data suggests that while non-inferior in preventing post-operative bile leak and BDI, utilisation of CTC leads to an increased risk of conversion to open surgery relative to IOC, as well as the need to perform subtotal cholecystectomies. As such, our data does not recommend the routine use of CTC in LC. It is feasible that a selective policy for CTC such as in patients who are expected to be technically challenging as a result of body habitus or previous surgery may be effective. However, more research needs to be undertaken on the subject.

The data above indicates a benefit to performing peri-operative imaging with either CTC or IOC as an adjunct to LC, and supports its routine use. IOC and CTC reduce rates of BDI and post-operative bile leak. This topic remains controversial, with strong supporters [2–4] and detractors [5–7]. A number of studies [10, 11] have suggested the link between aberrant biliary anatomy and incidence of bile duct injuries, and that identification of biliary anatomy may mitigate this risk. Other purported benefits include accuracy in diagnosis of choledocholithiasis, its negligible risk, and relatively low monetary and time expense if utilised appropriately [11]. This is directly contrary to other studies suggesting increased overall complications, and significant time and logistical investment [12].

The gallbladder is a dynamic organ, and concerns have been raised regarding the possibility of a change clinical situation in the two weeks prior to surgery, such as migration of stone into bile duct. However, no clinically significant instances of a missed stone was identified over the study period at this institution.

The use of CT cholangiography (CTC) prior to LC at our institution is currently dependent on the treating surgeon’s judgement, with varying practice from routine use to selective post-operative use as an adjunct in suspected choledocholithiasis. No guideline exists at present with regards to its use, and further research should be undertaken to assess its suitability.

### Effects on surgery

Despite the omission of IOC removing the need for radiographer availability, pre-operative CTC did not significantly reduce operative times relative to IOC, an effect suggested in other studies [12, 13]. However, not performing either modality resulted in decreased operative times. This may be related to caution exercised when performing LC once aware of the underlying anatomy via pre-operative CTC.

Length of stay is noticeably reduced in patients undergoing IOC relative to CTC. Without intraoperative confirmation local surgeon preference is for repeat LFTs prior to discharge home as an indicator for retained bile duct stones, which likely accounts for this disparity.

Incidence of BDI is generally quoted at 0.5% [4], while the overall BDI rate of this centre over the course is 0.37%. This rate increased significantly when peri-operative imaging was not performed. In absence of other contraindications, IOC is overall superior to CTC and as peri-operative imaging, and no evidence of clear benefits from utilising CTC were identified.

## Conclusion

Our data demonstrates evidence for the continued use of routine peri-operative biliary imaging with either IOC or CTC, with its benefits in reducing rates of post-operative bile leak and BDI. We recommend its continued use as part of regular LC surgery. However, there is a need to re-evaluate the practice of routine pre-operative CTC, given its relative inferiority to routine IOC in preventing conversion to open surgery and subtotal cholecystectomy. While instances of where a difficult IOC may occur, and it may not be feasible, a routine approach CTC is non-inferior at best, and likely to be detrimental in light of the findings above. Further research should be undertaken to evaluate criteria for a selective protocol for CTC.

## Data Availability

The datasets used and analysed during the current study are available from the corresponding author on reasonable request.
